# Randomized controlled trials of multi-sectoral programs: Lessons from development research

**DOI:** 10.1016/j.worlddev.2019.104822

**Published:** 2020-03

**Authors:** Agnes R. Quisumbing, Akhter Ahmed, Daniel O. Gilligan, John Hoddinott, Neha Kumar, Jef L. Leroy, Purnima Menon, Deanna K. Olney, Shalini Roy, Marie Ruel

**Affiliations:** a International Food Policy Research Institute (IFPRI), Washington DC, United States; b Cornell University, Ithaca, NY, United States

## Abstract

Development is a multi-faceted process; achieving development goals thus requires a multi-sectoral approach. For over two decades, our research group of economists and nutritionists has designed and implemented randomized trials to assess the effectiveness of multisectoral programs in improving nutrition, food security, and other measures of well-being, largely at the request of developing country governments, development partners, and non-governmental organizations. Our approach addresses three perceived pitfalls of RCTs: the “black box” nature of RCTs, limited external validity, and challenges in translation of results to impacts at scale. We address these concerns by identifying and assessing programmatic pathways to impact with quantitative and qualitative methods; studying similar programs implemented by different organizations across various settings; and working closely with implementing partners in the design, research, and dissemination processes to inform adaptation and scale-up of programs and policies.

## 1 Introduction

Development is a multi-faceted process; achieving development goals thus requires a multi-sectoral approach. For over two decades, our research group of economists and nutritionists at the International Food Policy Research Institute (IFPRI) has designed and implemented randomized controlled trials (RCTs) to assess the effectiveness of multi-sectoral social protection and agricultural interventions undertaken largely at the request of governments, development partners, and non-governmental organizations. The well-deserved Nobel Prize in Economics awarded to Abhijit Banerjee, Esther Duflo, and Michael Kremer revived a heated debate regarding the power and limitations of RCTs. Our approach to evaluations of multi-sectoral development programs addresses three perceived pitfalls of RCTs: the “black box” nature of the relationship between interventions and their impacts, limited external validity of findings, and challenges in translation of results to impact at scale.

## 2 Unpacking the “black box”

RCTs have been criticized as being a “black box”: they answer the question of whether the intervention had an impact, but do not inform on how impact was achieved. Going beyond the black box starts with developing a conceptual framework that maps hypothesized pathways of impact between the intervention and key outcomes (Olney et al., 2019). Program impact pathway frameworks are informed by the theory of change envisioned by implementers for a given program model and by evidence-based findings on behavioral, biological, and other plausible mechanisms.

For example, to understand whether – and how – nutrition- and gender-sensitive homestead food production programs with behavior change communication (BCC) improved child nutrition, we carried out a cluster-RCT to test the impacts of the program on maternal and child nutrition outcomes and used mixed methods, including process evaluations, to assess program impact pathways. We worked with Helen Keller International to develop the program’s hypothesized pathways of impact (Olney et al., 2013) and used survey data and process evaluations, respectively, to measure program impacts on “intermediate outcomes” along the pathways (quantitatively) and to understand operational issues (qualitatively). Exploration of operational issues drew on interviews with implementers and participants to understand their perceptions of barriers and facilitators: for program implementers to achieve optimal implementation, and for program participants to achieve uptake of the program in terms of utilization of services and adoption of recommended practices. Findings showed that the program reduced child anemia and wasting. The confirmed pathways of impact, which supported the plausibility of findings, included improvements in women’s agricultural production, empowerment, health and nutrition knowledge, and infant and young child feeding and care practices (Olney, Pedehombga, Ruel, & Dillon, 2015).

Mixed-method analyses of impact pathways are also useful for unveiling unintended consequences of programming. The Transfer Modality Research Initiative (TMRI), an experimental study designed by IFPRI and implemented by the World Food Programme in Bangladesh, randomized provision of food or cash transfers to poor rural women, alongside nutrition BCC. It led to sustained post-program reductions in participant women’s experience of intimate partner violence, an unintended benefit of the program. Drawing on a conceptual framework linking cash transfers to reductions in intimate partner violence (Buller et al., 2018) and using mixed methods, we found evidence suggesting these impacts emerged through increases in women’s social capital and empowerment, leading to sustained changes along three pathways: improvements in women’s bargaining power within their relationships, increases in men’s costs of perpetrating violence, and improvements in household economic security and emotional well-being (Roy, Hidrobo, Hoddinott, & Ahmed, in press).

## 3 External validity

Unlocking the black box contributes to understanding the external validity of our findings, i.e. the extent to which an intervention is expected to have similar effects in a different setting (Bates & Glennerster, 2017). Many of our impact evaluations teach us not merely “what happened,” but what conditions allowed the underlying mechanisms to play out. Although which mechanisms are affected in a given setting may not generalize to another, the conceptual framework itself is likely to have wider application and policy relevance across many settings.

We also generate information on external validity by conducting RCTs of similar interventions in different contexts. We worked with HarvestPlus to study the adoption of biofortified orange sweet potato (OSP) using comparable randomized experiments in two different country contexts, Mozambique and Uganda (de Brauw et al., 2018). The program aimed to promote adoption of OSP as a staple crop to increase vitamin A intake. The intervention provided access to OSP vines for planting, agriculture training, and nutrition training over a two-year period. Sweet potatoes were the primary staple food crop in Uganda, whereas in Mozambique, sweet potatoes were secondary staples. Mozambique has one rainy season, while Uganda has two. Thus, OSP vines had to be provided annually to farmers in Mozambique but only once in Uganda, while the models of training intensity for extension and nutrition were identical. Despite these differences in context, the interventions led to similar impacts in both countries, including adoption by 61–68% of farmers and a doubling of vitamin A intakes in children.

IFPRI also worked with the World Food Programme to evaluate the relative impact of cash transfers, food rations, and food vouchers on household food security and other outcomes in Ecuador, Uganda, Niger, and Yemen. RCTs were used to compare these modalities; the timing, frequency, and value of transfers were equalized to the extent possible across modalities. Cash (Ecuador, Uganda, Yemen) or vouchers (Ecuador) were more effective than food rations at improving household dietary diversity (Hidrobo et al., 2014, Gilligan and Roy, 2016, Schwab, 2019). Only in Niger, where only a limited number of foods were available in local markets, was food more effective than cash transfers (Hoddinott, Sandström, & Upton, 2018). Cost data showed that cash transfers were cheaper to deliver, strengthening the evidence for the cost effectiveness of cash in improving dietary diversity in settings where food markets are accessible.

A third example was the evaluation of a food-assisted maternal and child health and nutrition program using RCTs in Guatemala and Burundi. The program targeted women and their children during the first 1000 days and included food rations, health services strengthening, and BCC. While both settings were characterized by very high levels of stunting (about 60%), food insecurity was a severe problem only in Burundi. The program reduced the prevalence of stunting in both settings, but also contributed to postpartum weight retention in the already overweight study population in Guatemala (Leroy et al., 2018, Leroy et al., 2019). This finding underscores the importance of carefully analyzing context when designing programs to prevent unintended negative consequences on targeted populations.

## 4 Translating results to impact at scale

A hallmark of our approach to evaluation is engagement with project partners and other local stakeholders throughout the research process, to ensure that our research not only produces global public goods but also helps inform better program design in the country setting itself. In our experience, early collaboration best enables the design of evaluations that prioritize stakeholders’ questions and earn their buy-in, increasing the chances that findings are taken up and used to guide scale-up of a program model. Although we take this approach with the range of methods we use for causal inference in impact evaluations, it is particularly important for RCTs. IFPRI’s evaluation of Mexico’s PROGRESA over two decades ago is an example of one of our earliest efforts to build an engaged RCT-based evaluation of a complex national program being implemented at scale (Skoufias, 2005). IFPRI staff were part of a team that worked closely with PROGRESA staff throughout the development, design, implementation, and dissemination of the research. Insights from the PROGRESA evaluation were eventually used to design similar large-scale cash transfer programs in Mexico, Brazil, and Honduras.

The substantial presence of our research teams in offices in Sub Saharan Africa and South Asia also helps continuous engagement throughout the research process and often supports policy adoption and scale-up. An example of the impacts of our long-term presence and engagement in Bangladesh is the collaborative RCT we carried out with the Bangladesh Ministry of Agriculture (BMoA) on the Agriculture, Nutrition, and Gender Linkages (ANGeL) Project. The evaluation showed that combining agricultural production, nutrition knowledge, and gender sensitization trainings most effectively improved production and consumption diversity, farmers’ income, and women’s empowerment. Dissemination and continued engagement around these results motivated the BMoA to scale up the program nationally—the first time any government agency in Bangladesh used rigorous research-based results to justify a national program. The new program is now being rolled out as a large-scale RCT designed in collaboration with IFPRI, the first such experimental study of a national program in Bangladesh.

## 5 Conclusions

Our experience illustrates how well-designed pragmatic RCTs, informed by theory, process evaluations, qualitative research, and collaborative engagement between researchers and implementers from the design phase through the interpretation and utilization of results can generate evidence that helps partners design and implement successful multi-sectoral development programs at scale. This type of collaboration helps program implementers learn whether their good intentions have the envisioned benefits, while bringing together the tools of research with the voices of those whose lives are affected to advance our collective understanding of what works to improve the health, nutrition, and well-being of the poor.
